# Inverted duplication, triplication and quintuplication through sequential breakage‐fusion‐bridge events induced by a terminal deletion at 5p in a case of spontaneous abortion

**DOI:** 10.1002/mgg3.965

**Published:** 2019-09-02

**Authors:** Hongyan Chai, Brittany Grommisch, Autumn DiAdamo, Jiadi Wen, Pei Hui, Peining Li

**Affiliations:** ^1^ Clinical Cytogenetics Laboratory Department of Genetics Yale University School of Medicine New Haven CT USA; ^2^ Department of Pathology Yale University School of Medicine New Haven CT USA

**Keywords:** breakage‐fusion‐bridge, inverted duplication, terminal deletion at 5p

## Abstract

**Background:**

Integrated chromosome, fluorescence in situ hybridization (FISH) and array comparative genomic hybridization (aCGH) analyses have been effective in defining unbalanced chromosomal rearrangements. Discordant chromosome and aCGH results are rarely reported.

**Methods:**

Routine cytogenomic analyses and literature review were performed in the study of a case from products of conception (POC).

**Results:**

Chromosome and FISH analysis revealed a mosaic pattern consisting of a primary aberration of an inverted duplication of 5p and derived secondary and tertiary aberrations from sequential triplication and quintuplication of 5p, respectively. The aCGH analysis detected only a 1.521 Mb terminal deletion at 5p15.33 with no other pathogenic copy number variants in the genome. This mosaic karyotypic pattern likely resulted from chromosome instability induced by sequential breakage‐fusion‐bridge events during in vitro cell culture. A review of literature found heterogeneous distal deletion and inverted duplication of 5p in prenatal and pediatric cases.

**Conclusion:**

This is the first case reported in POC with a unique mosaic pattern and discordant chromosome and aCGH results. Caution should be applied in reporting and interpreting these discordant results and further analysis for underlying mechanism should be considered.

## 1 INTRODUCTION

Integrated chromosome, fluorescence in situ hybridization (FISH), and array comparative genomic hybridization (aCGH) analyses have been used in prenatal diagnosis and revealed a spectrum of cytogenomic abnormalities from products of conception (POC) (Chai et al., 2019; Zhou, Wu, Amato, DiAdamo, & Li, 2016). Chromosome analysis has been effective in detecting numerical and structural chromosomal abnormalities, while aCGH has the advantage to define genomic coordinates and gene content from unbalanced structural chromosomal abnormalities and to detect submicroscopic pathogenic copy number variants (Wei et al., 2012; Xu, DiAdamo, Grommisch, & Li, 2013; Zhang, Xu, Seashore, & Li, 2012). However, in a recent study on a case of POC, a mosaic pattern derived from an inverted duplication, triplication and quintuplication of 5p was detected by karyotyping and FISH, but only a terminal deletion at 5p15.33 was detected by aCGH. The discordant results revealed a chromosomal instability likely induced by sequential breakage‐fusion‐bridge events observed in metaphase cells from in vitro cell culture. Interpretation of these discordant results and its clinical implications were discussed.

## 2 CASE PRESENTATION AND CYTOGENOMIC RESULTS

A 38‐year‐old woman, who was gravida 2, para 1, at 6+ weeks presented with missed abortion by last menstrual period and ultrasound measurements. After informed consent, the patient underwent dilation and curettage with suction. POC were obtained and sent to pathology and clinical cytogenetics laboratories. The curettage specimen showed the presence of chorionic villi with mild hydropic to fibrotic changes and absence of significant size enlargement, cistern formation or villous trophoblastic hyperplasia. Chromosome, FISH, and aCGH analyses were performed following standardized procedures in the cytogenetics laboratory.

Chromosome analysis was performed on cultured fibroblast cells from the dissected POC villi. Of a total 50 metaphase cells examined, a mosaic pattern consisting of three types of 5p rearrangements was noted. The primary type (type I) with a derivative chromosome 5 from a terminal deletion at 5p15.33 and an inverted duplication of 5p15.33p13.3 was noted in 19 cells, the secondary type (type II) featuring a derivative chromosome 5 from type I aberration with further triplication of 5p15.33p13.3 was noted in 24 cells, and the tertiary type (type III) featuring a derivative chromosome 5 from a quintuplicate (qtp) of 5p15.1p13.3 was noted in seven cells. To determine if these complex patterns of structural chromosome rearrangements were derived from a balanced chromosomal rearrangement in one of the parents, follow up parental chromosome analysis was recommended. The mother showed a normal female karyotype and the father rejected chromosome analysis.

A FISH test was performed on cultured villi cells using dual color probes for the subtelomeric 189N21 locus at 5p15.33 (RH120167, chr5:2.222–2.422 Mb) and the *TAS2R1* gene at 5p15.31 (chr5:9.6 Mb) (Cytocell Inc.). Of the 100 nuclei examined, 3% had the normal pattern of two signals for each probe and 97% showed an abnormal pattern of three signals for the 189N21 probe and four signals for the *TAS2R1* probe. Further examination of FISH signal patterns on abnormal metaphases observed the presence of interstitial *TAS2R1*/189N21/*TAS2R1* signals in the type I inverted duplicated chromosome 5, of distal 189N21/*TAS2R1* signals and interstitial *TAS2R1*/189N21/*TAS2R1* signals in the type II derivative chromosome 5, and of interstitial *TAS2R1*/189N21/*TAS2R1* signals in the type III derivative chromosome 5. The abnormal FISH result on cultured interphase cells showed a signal pattern consistent with the type II abnormal karyotype on metaphase cells, given that the 189N21 signal near the break‐fusion locus could be viewed as one signal. The type I and type III patterns were not observed in cultured interphase cells.

**Figure 1:**
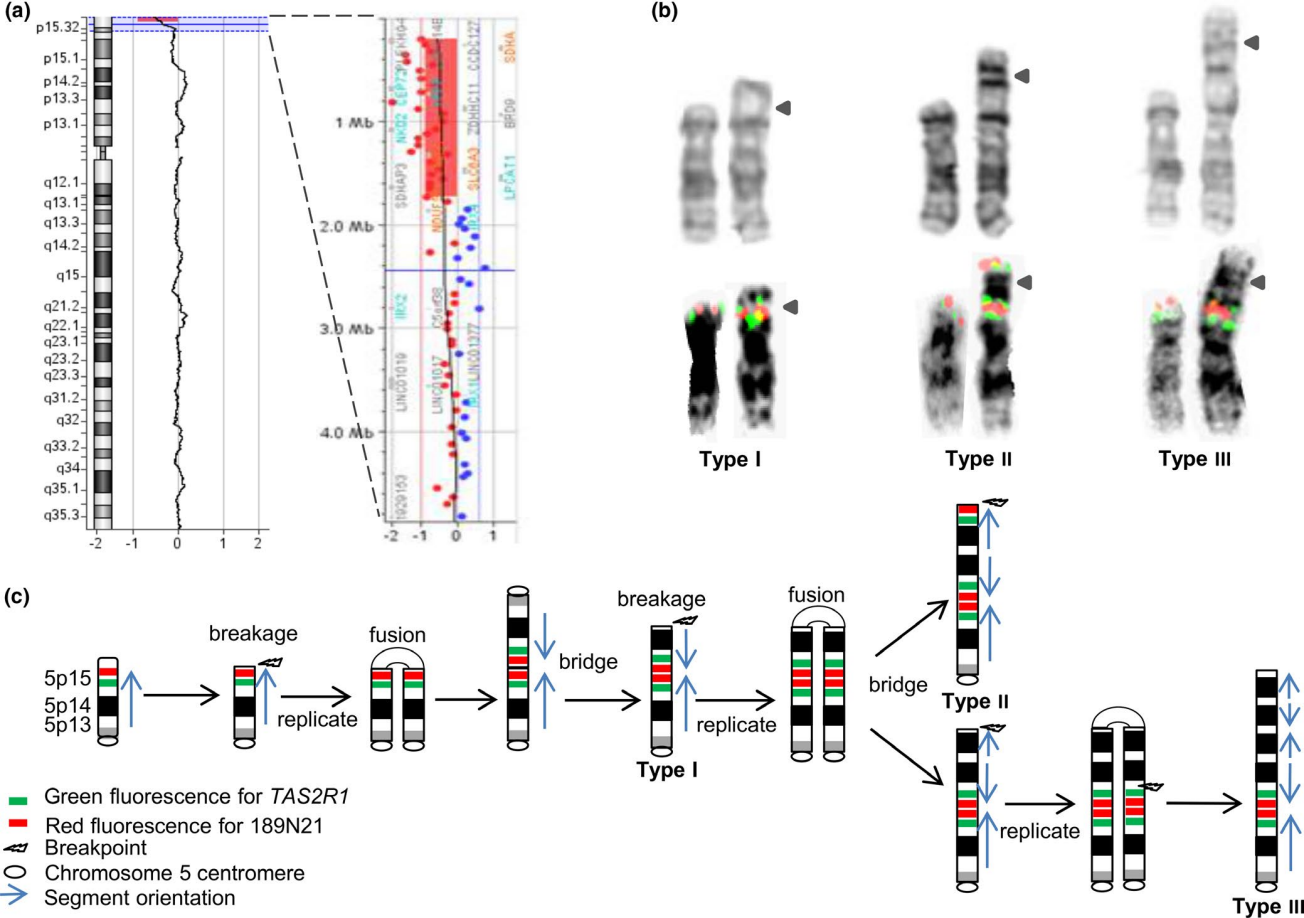
(a) An aCGH analysis showing a 1.521 Mb deletion at 5p15.33 (chr5:204,737–1,726,099). (b) Three types of 5p rearrangements revealed by chromosome G‐banding (the upper row) and metaphase FISH results (the lower row). Probes for the *TAS2R1* gene and the 189N21 locus are labeled green and red, respectively. (c) A diagram showing a sequential breakage‐fusion‐bridge process in the formation of a mosaic pattern with different 5p rearrangements

DNA extracted from the POC villi tissue was subjected to aCGH analysis on SurePrint G3 Human CGH Microarray 8x60K (Agilent, Santa Clara, CA). The result revealed an XY male with a 1.521 megabase (Mb) deletion at 5p15.33 (chr5:204,737–1,726,099) as the sole anomaly. This deletion includes 24 genes from *PLEKHG4B* to *SDHAP3*. A repeat analysis using Affymetrix OncoScan array confirmed this aCGH result (data not shown). This microarray result indicated the presence of a subtelomeric terminal deletion at 5p15.33, but did not reveal any segmental duplication at 5p or gain or loss of genetic material from other chromosomes (Figure 1). The 189N21 and *TAS2R1* probes are located outside of this deletion and the 3% interphase cells with a normal FISH pattern could carry this deletion.

Combined chromosome and FISH results showed a karyotypic evolution from type I to types II and III through sequential breakage‐fusion‐bridge events (Figure 1). The detailed designation for this mosaic pattern by An International System for Human Cytogenetic Nomenclature 2016 (McGowan‐Jordan, Simons, & Schmid, 2016) is: mos 46,XY,der(5)del(5)(pter‐>p15.33)dup(5)(p13.3‐>p15.33::p15.33‐>qter)[19]/46,XY,der(5)del(5)(pter‐>p15.33)trp(5)(p15.33‐>p13.3::p13.3‐>p15.33::p15.33‐>qter)[24]/46,XY,der(5)del(5)(pter‐>p15.33)qtp(5)(p15.1‐>p13.3::p13.3‐>p15.1::p15.1‐>p13.3::p13.3‐>p15.33::p15.33‐>qter)[7].

## 3 DISCUSSION

**Figure 2:**
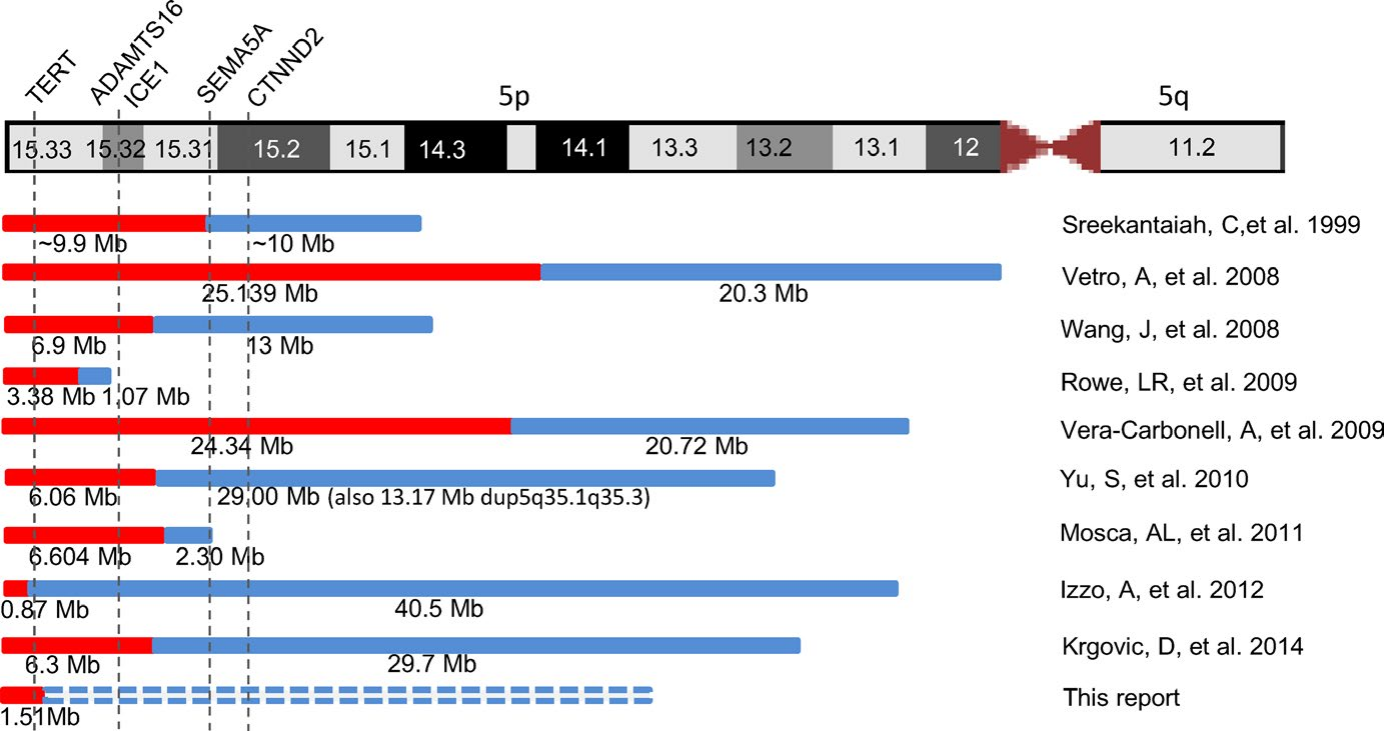
A diagram showing the terminal deletions and inverted duplications of 5p in reported cases and the present case. Red bar is for deletion and blue bar is for inverted duplication. The candidate genes for Cri‐du‐Chat syndrome from 5p deletions are shown on the top

Distal deletions of 5p cause Cri‐du‐Chat syndrome (OMIM #123450) with a cat‐like cry in infancy, dysmorphic facial features, microcephaly and intellectual disability. The critical region for the cat‐like cry was mapped to a 1 Mb interval at 5p15.32 encompassing a candidate gene *ICE1* (OMIM *617958), which regulates small nuclear RNA transcription; the *TERT* (OMIM *187270) gene at 5p15.33 and the *SEMA5A* (OMIM *609297) and *CTNND2* (OMIM *604275) genes at 5p15.31p15.2 were considered candidate genes for autistic and cognitive phenotypes (Zhang et al., 2016). Review of literature found nine cases with a distal deletion and an inverted duplication of 5p. Two newborns and two pediatric cases showed high pitch cry at birth, dysmorphic features, and development delays (Krgovic, Blatnik, Burmas, Zagorac, & Kokalj Vokac, 2014; Sreekantaiah, Kronn, Marinescu, Goldin, & Overhauser, 1999; Vera‐Carbonell et al., 2009; Wang et al., 2008). These observations supported the mapped critical region for a cat‐like cry (Figure 2). Three cases were detected prenatally and showed abnormal findings by ultrasound or postmortem examination (Izzo et al., 2012; Mosca et al., 2011; Vetro et al., 2008). Two cases showed only cytogenetic results (Rowe et al., 2009; Yu & Graf., 2010). Of these nine cases, the distal deletions of 5p ranged from 0.87 Mb to 25 Mb and the segmental duplications ranged from 1.07 Mb to 40.5 Mb (Figure 2). The deletion in this POC case includes the *TERT* gene, but not the *ICE1, SEMA5A* and *CTNND2* genes.

Terminal deletions can cause chromosome instability by initiating a U‐type exchange and a breakage‐fusion‐bridge process (Murnane, 2012). The U‐type exchange was indicated as the most frequent mechanism for inverted duplication with terminal deletion rearrangements (Rowe et al., 2009; Yu & Graf., 2010). This mechanism involves firstly a double‐strand breakage at the subtelomeric region for a terminal deletion, secondly a fusion by nonhomologue end join (NHEJ) to form a dicentric chromosome, and thirdly a chromatin bridge between daughter cells in mitosis which will be followed by subsequent asymmetric breakage of the dicentric intermediate and resolved to have an inverted duplication (Maciejowski, Li, Bosco, Campbell, & de Lange, 2015). This breakage‐fusion‐bridge process occurred mostly de novo in the prezygotic meiosis stage and the resulting terminal deletion and inverted duplication in chromosome 5 were relatively stable in reported cases. The present case showed a terminal deletion encompassing the *TERT* gene and clearly a chromosome instability through a breakage‐fusion‐bridge process in the formation of a mosaic pattern with sequential duplication, triplication and quintuplication of 5p. The *TERT* (telomerase reverse transcriptase) gene encoded the rate‐limiting component of telomerase complex essential for telomere length maintenance and sustained cell proliferation (Zhang et al., 2003). It should be noted that the G‐band pattern in the fusion region of the type I derivative chromosome 5 showed enlarged light band material which might be amplified repetitive sequences undetectable by aCGH.

The discordant results from chromosome, FISH and aCGH in this case pose a challenge in interpreting and reporting. In the mosaic pattern, the type I aberration with a terminal deletion and an inverted duplication of 5p could be the initial aberration. The related types II and III were derived from type I. Interphase FISH showed that the majority of cells had a duplication of subtelomeric 189N21 locus at 5p15.33 and a triplication of *TAR2R1* gene at 5p15.31, which was consistent with type II derivative chromosome 5 with a triplication of 5p (Figure 1). The result from aCGH showed a terminal deletion at 5p15.33 while the large inverted duplications in the 5p were not detected by aCGH. Discordant chromosome and microarray results were rare in prenatal and postnatal cytogenetics practice, but had been reported in miscarriages (Shah et al., 2017). Discordances in miscarriages were thought due to maternal cell contamination, balanced chromosome rearrangements, polyploidy, and placental mosaicism. The mosaic pattern in the present case cannot be explained by these situations. In a newborn with a ring chromosome 13, discordant chromosome and microarray results were noted in repeated studies (Kaylor et al., 2014). Initial chromosome analysis found a double ring chromosome 13 and microarray showed a large duplication and distal deletion in chromosome 13. A repeat chromosome analysis found a mosaic pattern of ring chromosome 13, double ring 13, and monosomy 13, but microarray analysis showed only the distal deletion. It was postulated that mitotically unstable double rings had a high rate of cell death. Dicentric ring chromosomes were also detected with distal deletion and segmental duplication by the breakage‐fusion‐bridge process through cell cycles (Hu, Chai, Shu, & Li, 2018; Rowe et al., 2009; Xu et al., 2013; Zhang et al., 2012). The discordant chromosome and microarray results in the present case is likely due to stringent in vivo selection by high rate of cell death for cells with derivative chromosomes 5 and the release of this selection during in vitro cell culture.

In conclusion, prenatal and postnatal cases with an inverted duplication induced by a terminal deletion at 5p have been observed as heterogeneous cytogenomic abnormalities. During in vitro cell culture, chromosomal instability by sequential U‐type exchange and breakage‐fusion‐bridge process could result in complex duplication, triplication and quintuplication of 5p in chromosome analysis. The cytogenetic result and its clinical significance should be interpreted with caution. Further study is needed to understand the in vivo selection mechanism of dicentric chromosome segregation through mitosis.

## CONFLICTS OF INTEREST

The authors declare no conflict of interest. There were no prestudy requirements on the patient's specimens and clinical indications and no poststudy interaction and intervention with the patients. This clinical report was a retrospective study and deemed exempted from Institutional Review Boards (IRB) approval and waiver of consent based on the policy of Yale University IRB.
